# Inter-rater reliability of malaria parasite counts and comparison of methods

**DOI:** 10.1186/1475-2875-8-267

**Published:** 2009-11-25

**Authors:** Katherine M Bowers, David Bell, Peter L Chiodini, John Barnwell, Sandra Incardona, Seiha Yen, Jennifer Luchavez, Hilary Watt

**Affiliations:** 1 Hospital for Tropical Diseases, WC1E 6JB, UK; 2 Foundation for Innovative Diagnostics (FIND), Geneva, Switzerland (formerly WHO-Regional Office for the Western Pacific, Manila, Philippines); 3 London School of Hygiene and Tropical Medicine, WC1E 7HT, UK; 4 CDC, Buford Highway, Atlanta, Georgia, USA; 5 Laboratory of Molecular Epidemiology, Institut Pasteur of Cambodia, BP 983 Phnom Penh, Cambodia; 6 Research Institute for Tropical Medicine, Department of Health Compound, FILINVEST Corporate City, Alabang, Muntinlupa City, 1781, Philippines

## Abstract

**Background:**

The introduction of artemesinin-based treatment for falciparum malaria has led to a shift away from symptom-based diagnosis. Diagnosis may be achieved by using rapid non-microscopic diagnostic tests (RDTs), of which there are many available. Light microscopy, however, has a central role in parasite identification and quantification and remains the main method of parasite-based diagnosis in clinic and hospital settings and is necessary for monitoring the accuracy of RDTs. The World Health Organization has prepared a proficiency testing panel containing a range of malaria-positive blood samples of known parasitaemia, to be used for the assessment of commercially available malaria RDTs. Different blood film and counting methods may be used for this purpose, which raises questions regarding accuracy and reproducibility. A comparison was made of the established methods for parasitaemia estimation to determine which would give the least inter-rater and inter-method variation

**Methods:**

Experienced malaria microscopists counted asexual parasitaemia on different slides using three methods; the thin film method using the total erythrocyte count, the thick film method using the total white cell count and the Earle and Perez method. All the slides were stained using Giemsa pH 7.2. Analysis of variance (ANOVA) models were used to find the inter-rater reliability for the different methods. The paired t-test was used to assess any systematic bias between the two methods, and a regression analysis was used to see if there was a changing bias with parasite count level.

**Results:**

The thin blood film gave parasite counts around 30% higher than those obtained by the thick film and Earle and Perez methods, but exhibited a loss of sensitivity with low parasitaemia. The thick film and Earle and Perez methods showed little or no bias in counts between the two methods, however, estimated inter-rater reliability was slightly better for the thick film method.

**Conclusion:**

The thin film method gave results closer to the true parasite count but is not feasible at a parasitaemia below 500 parasites per microlitre. The thick film method was both reproducible and practical for this project. The determination of malarial parasitaemia must be applied by skilled operators using standardized techniques.

## Background

With the introduction of artemisinin-based treatment for falciparum malaria there has been a shift away from symptom-based diagnosis to diagnosis based upon the detection of malaria parasites. Rapid non-microscopic diagnostic tests (RDTs) are one way in which this may be achieved, but light microscopy has a central role in parasite identification and quantitation, is necessary for monitoring the accuracy of RDTs, and remains the main method of parasite-based diagnosis in large clinic and hospital settings.

Although molecular diagnostic methods have made substantial progress and are undoubtedly the most sensitive techniques for the diagnosis of malaria, for most clinical laboratories, blood film examination remains the final arbiter and allows determination of the parasite densities. There are however, both different blood film and different counting methods used for this purpose which raises questions regarding which gives the most accurate figure and which is the most reproducible.

The World Health Organization has prepared a proficiency testing panel containing a range of malaria-positive blood samples of known parasitaemia, to be used for the assessment of commercially available malaria RDTs. Before doing so, a comparison was made of the established methods for parasitaemia estimation to determine which would give the least inter-rater and inter-method variation.

## Aims

To compare different methods of counting malaria parasites, both in terms of inter-rater reliability of the different methods, and also to assess any relative bias between the methods.

## Methods

The blood films were collected and prepared with the approval of the Research Ethics Committees of The Ministry of Health, Cambodia, as part of the collection of samples and development of methods for monitoring malaria rapid diagnostic test quality in a joint project with the World Health Organization.

The study sites were the Research Institute for Tropical Medicine, Alabang, the Philippines (RITM); Institut Pasteur, Phnom Penh, Cambodia (IP); the Centers for Disease Control and Prevention, Atlanta, Georgia, United States (CDC); and the Hospital for Tropical Diseases, London, UK (HTD).

Thirty-one blood samples containing *Plasmodium falciparum *were collected on a field trip to Cambodia in September 2004.

Study participants were enrolled during a high transmission season in two regions (Kampot and Pailin). The purpose and methods of the study were explained in the regional dialect orally and in written form. Informed consent was obtained from the study participant or the legal guardian. An independent witness also signed each consent form. Patients with a history of fever had a malaria RDT performed. Any patient, over the age of seven with a positive RDT, without serious malaria symptoms and without treatment with anti-malarial drugs within the past three weeks was interviewed and enrolled. Thick and thin smears [1] were made from fingerpick samples for analysis at Institut Pasteur of Cambodia (IP). Ten to fifteen ml of blood was collected in EDTA tubes. The blood was kept on ice and transported to the laboratory at the end of the day in an air-conditioned vehicle. Earle and Perez [2] and thin blood films were made at IP from an EDTA tube on receipt of the samples and a total white cell count and red cell count was performed the next day using a Cell Counter (Cell-Dyn 3200 ABBOTT) on one of the refrigerated EDTA tubes. DNA was extracted from 0,2 ml EDTA blood, using the QIAamp DNA blood kit (QIAGEN, Germany). A species-specific nested PCR method, based on 18S rRNA gene amplification, was used to determine the infecting species, as previously described [3].

The Cambodian microscopists were prequalified by the WHO malaria accreditation course[4]. A microscopist from the Cambodia National Malaria Control Centre prepared the slides in collaboration with a reference microscopist from HTD; the latter also monitored the counting of parasites in the field laboratory and later repeated the parasite counts at HTD on a separate stained slide. Repeat counts were only performed if the results showed a >20% discrepancy between methods or readers. Extra slides were made and stained from the EDTA sample collected in the field. The extra slide sets were sent to CDC, HTD and RITM for separate evaluation. The slides were mounted in DPX and a coverslip was applied before the slides were sent to the different laboratories. Only the reference reader had access to all the counts for analysis of the results at the end of the study, and microscopists were otherwise blinded to each other's results.

**Figure 1:**
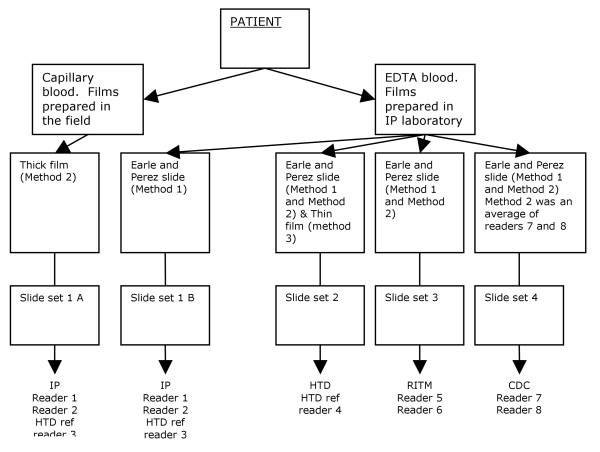
**Diagram of slide sets and readers**.

Counts were performed on Earle and Perez slides using the method described by Earle and Perez [2] and were analysed as Method 1. The number of parasites found in fields containing 500 white blood cells was performed on Earle and Perez slides or the thick film (see Figure 1). The total number of parasites per 500 white cells was adjusted for the true white cell count and was designated Method 2

Method 3 was performed on the thin film and the number of parasites per 5000 erythrocytes was adjusted for the total erythrocyte count. A 1 mm^2 ^× 1 mm^2 ^grid divided into 10 by 10 squares was used to perform the counts in Method 3.

Seven raters at four different sites counted the parasites (Figure 1). Each rater counted separately each sample using two different methods, the Earle and Perez and the thick film count. Since one of the seven raters counted the parasites twice on each sample (once at each of IP and HTD, there were eight counts in total of the parasites, on each sample, for the Earle and Perez method. However, for the thick film count method, at CDC, the individual counts for the two raters were averaged and only this mean value was available for analysis, therefore only seven counts were available per sample (including two from the same rater at different sites, and another representing an average of two raters). There was a slight variation between sites in how the thick film count was undertaken - capillary blood was used in Cambodia, and EDTA blood was used for the other sites. For a third method, the thin cell count method, a single rater counted the parasites once only, from separate slide preparations of the same blood samples.

Method 1 was that of Earle and Perez; method 2 counted parasites against the known white cell count on a thick blood film; and method 3 counted parasites against the known red blood cell count on a thin blood film.

Any samples found to contain more than one species of malaria parasite (ie mixed) by microscopy at any of the sites were removed from the analysis of the data. Samples that were found to be mixed infections by PCR but not by microscopy were still included in this study [3]. The parasite counts were analysed at The London School of Hygiene and Tropical Medicine Statistics Department for variation between readers and between methods at the different sites. IP, RITM and HTDL counted sexual stage parasites (gametocytes) as well as asexual stage parasites, whilst CDC counted only asexual stages. Sexual stages were found in only 16 samples and were present in low numbers, so statistical data was performed only on asexual stages.

### Statistical analysis

Firstly, counts were plotted to assess their distribution and scatter, and to assess the necessity of the log transformation. The log scale is used to analyse these counts, so that proportional differences, and not absolute differences, are assessed. Therefore, a halving represents the same size of difference as a doubling, but in the opposite direction. It also means that an increase from 500 to 1,500 in the parasite count is treated as a tripling, in comparison to a base of 500, whereas it is treated as a 1% increase, in comparison to a base of 100,000. The average reported is the geometric mean, which is calculated as exponential of the mean of the log counts. The geometric mean can also be calculated as the *n*^th ^root of the product of all the observations, where *n *is the sample size. Geometric standard deviations are quoted, which can be calculated as the exponential of the standard deviation of the logs. These geometric standard deviations are multiplicative standard deviations, e.g. one standard deviation above the average = (geometric mean) × (geometric standard deviation), and two standard deviations below the average = (geometric mean) ÷ (geometric standard deviation)^2 ^[5].

For Earle and Perez, and the thick film methods, inter-rater reliability was assessed. Firstly, Bland and Altman plots were drawn [6]. Each individual value was expressed relative to the geometric mean of the counts for that sample (the two separate counts that the one rater made were counted as separate counts at this stage). For the thick film count method, corresponding plots were also drawn, restricted to those sites where EDTA blood was used (5 raters at UK, Philippines and USA).

Analysis of variance (ANOVA) models, including each sample identifier as a fixed effect, were used to find the inter-rater reliability for the different methods. For each method of measurement, this was done in two stages. Firstly the within rater variance (W) was estimated from a model for the repeated counts from the one rater who estimated parasite counts twice on each sample. Secondly the between rater variance (B) was estimated via a model for the counts from different raters (using the average of the two counts for the one rater with two available, and using averages across raters where only these were available.).

For each method of measurement, the residual variance from the first model is an estimate of W. The residual variance from the second model is an estimate of a linear combination of B and W, with the linear combination differing between measures because different measures are aggregated in different ways. For Earle and Perez, the residual variance from this model is an estimate of (6.5 × W + 7 × B)/7 (because the mean of two measures is used for one of the seven raters). For the thick film method the analogous expression is (5 × W + 5.5 × B)/6 (because the mean of two measures is used for one of the raters and results from two more are averaged). For the thick film method, restricted to EDTA blood, the analogous expression is (3.5/4) × (W+B). By substituting for W from the first model, B was estimated for each measure. The quoted results are based on both within and between rater variance, (i.e. based on B+W), in order to reflect the reliability, when different raters count each sample only once. 95% reference ranges were found, relative to the geometric mean count for the specified sample, across all raters. 95% of counts are expected to lie within these reference ranges. The calculations are based on assuming a normal distribution for differences between each raters' log counts and the mean log count [calculated by exp(+/-1.96 × v(B+W))]. These assumptions of normality were broadly satisfied.

After assessment of inter-rater reliability, average counts were compared between methods of measurement, to see if there were any biases in average parasite counts from one method relative to another method. Again, Bland and Altman plots were used. Firstly, the geometric mean across all raters was taken for Earle and Perez and the thick film method. The relative difference between these averages (across raters) was plotted against the geometric means (over both methods) of these averages. The paired t-test was used to assess any systematic bias between the two methods, and a regression analysis was used to see if there was a changing bias with parasite count level. Corresponding analyses were used, to compare Earle and Perez with the thin film method, and then to compare the thick with the thin film methods. Note that for the thin film method, only a single count by one rater was available for each sample, so this was used in place of the geometric mean across raters.

## Results

**Figure 2:**
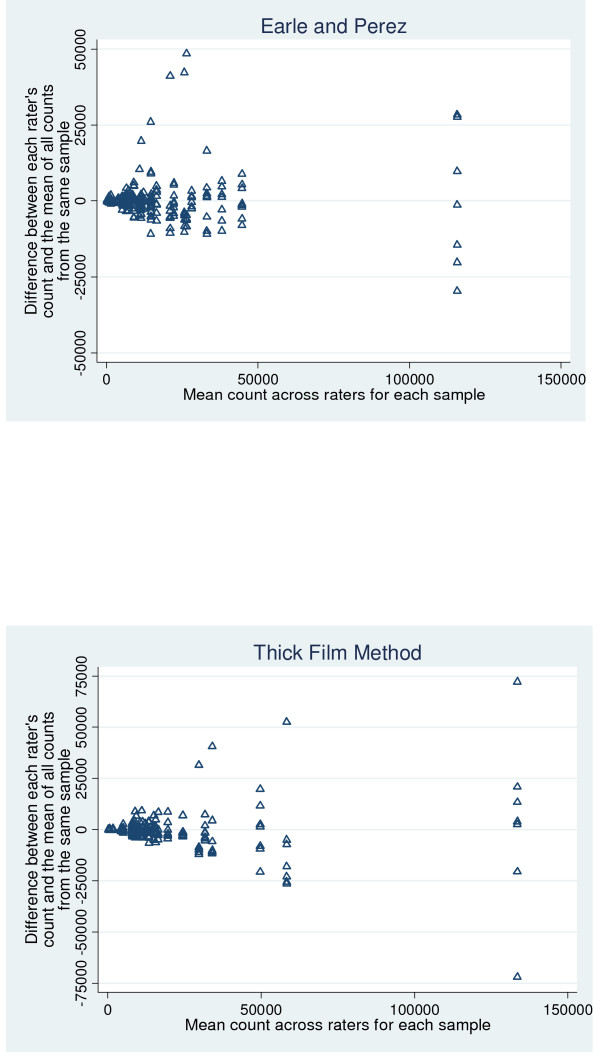
**Inter-rater reliability for each method, on original (*not*log) count scale**. (a) Earle and Perez method. x axis: Mean count across raters for each sample. y axis: Difference between each raters count and the mean of all counts from the same sample. (b) Thick film method. X axis: Mean count across raters for each sample. Y axis: Difference between each raters count and the mean of all counts from the same sample.

**Figure 3:**
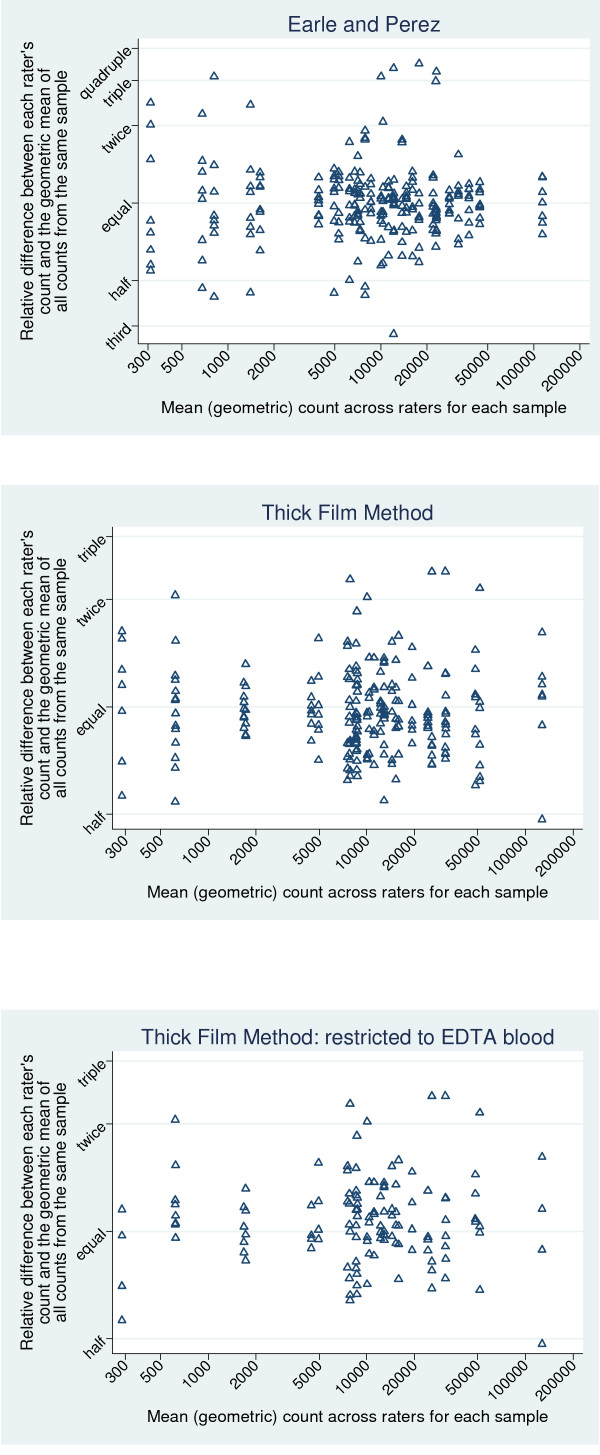
**Inter-rater reliability for each method, on log count scale, and therefore showing relative differences**. (a) Earle and Perez graph. X axis: Mean (geometric) count across raters for each sample. Y axis: Relative difference between each raters count and the geometric mean of all counts from the same sample. (b) Thick film method. X axis: Mean (geometric) count across raters for each sample. Y axis: Relative difference between each raters count and the geometric mean of all counts from the same sample. (c) Thick film method restricted to EDTA Blood. X axis: Mean (geometric) count across raters for each sample. Y axis: Relative difference between each raters count and the geometric mean of all counts from the same sample.

The samples included in this study differed substantially in their parasite counts. For Earle and Perez, the mean counts ranged from 366 to 116,000, and for the thick film method, the range was 304 to 134,000. Plots on the original scale (Figures 2a and 2b) show a cluster of points with lower parasite counts, and a few outlying values. The scatter of the differences (on the y-axis) tends to increase with increasing mean counts. Use of log scales (as shown in Figure 3) results in a more uniform scattering of the points on the graphs, and a scatter that is roughly constant with increasing count. The relatively constant scatter implies that use of the log scale, and associated relative differences, is an appropriate and useful way to summarize these data. The corresponding geometric means and geometric standard deviations (which represent the transformation of the results on log counts back onto the original scale) were, therefore, quoted.

### Repeatability of Earle and Perez method

The estimated between rater geometric SD (i.e. multiplicative SD) of the counts, based on one count per rater, is 1.46. This implies that 95% of raters will give individual counts between 48% (= 100% × 1.46^-1.96^) of the geometric mean count across all raters, for the specified sample, and 2.10 (= 100% × 1.46^1.96^) times this count.

### Repeatability of the thick film method

The estimated between rater geometric SD of the counts, based on one count per rater, is 1.36. This implies that 95% of raters will give individual counts between 54% of the geometric mean count across all raters, for the specified sample, and 1.84 times this count. Restricting our analyses to sites 2 to 4 only (i.e. where EDTA bloods were used), makes little difference to our results. Then, the estimated between rater geometric SD of the counts is 1.39, which implies that 95% of raters will give individual counts between 53% of the geometric mean across all raters, and 1.90 times the geometric mean.

### Intra-rater repeatability

The single rater, who counted all samples twice, gave very consistent counts for Earle and Perez (i.e. she agreed with herself very closely, far better, in general, than her agreement with the other raters, and their agreement with each other). However, this rater did not give consistent results for the thick film method; her repeated counts were about as similar to her initial counts as they were to other raters' counts. Hence, this implies that Earle and Perez has much better intra-rater reliability than the thick film method. However, it is impossible to know whether other raters would give similarly consistent results for Earle and Perez, but not for the thick film method, had they rated the samples twice. This difference may also be due to the fact that the exact same method was used twice by this rater, for Earle and Perez, whereas for the thick film method, she used capillary blood in Cambodia, and EDTA blood in the UK.

### Agreement between counts from Earle and Perez and the thick film method (Figure 4a)

**Figure 4:**
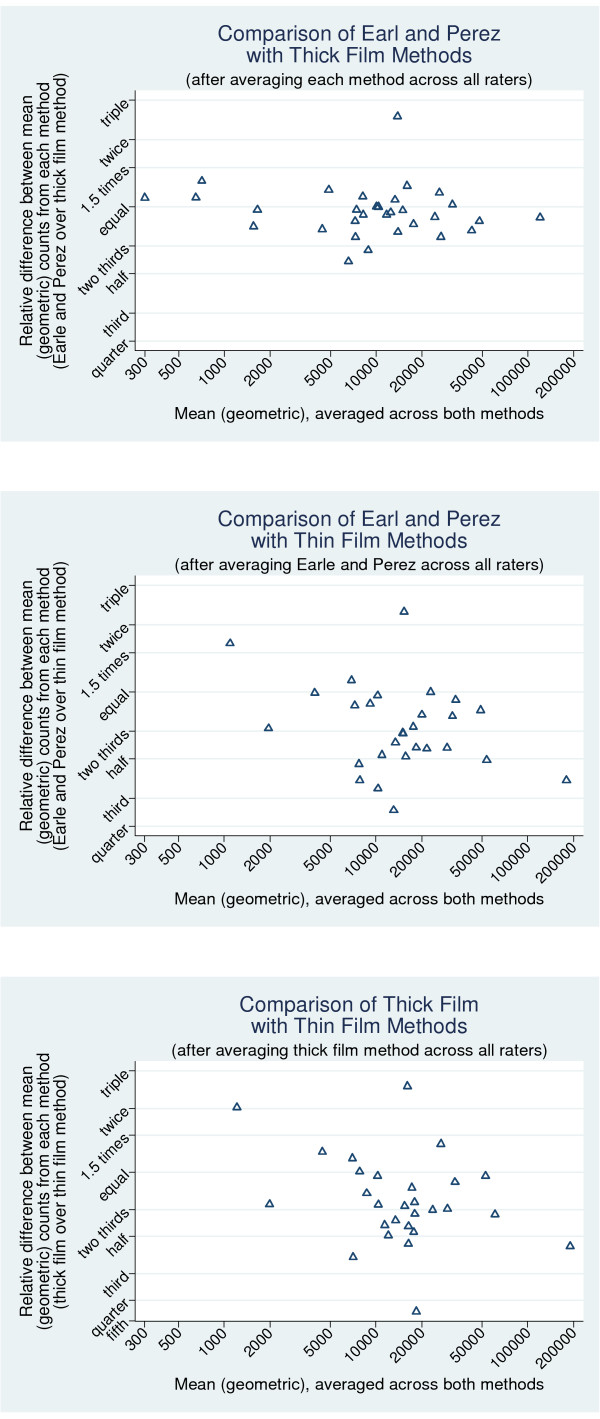
**Bland and Altman plots to demonstrate the presence of any bias between different methods of counting the parasite loads, on a log scale, to show relative differences**. (a) Comparison of Earle and Perez with thick film methods (after averaging each method across all raters). X axis: Mean (geometric) averaged across both methods. Y axis: Relative difference between mean (geometric) counts from each method (Earle and Perez over Thick film method). (b Comparison of Earle and Perez with Thick Film methods (after averaging Earle and Perez across all raters). X axis: Mean (geometric) averaged across both methods. Y axis: Relative difference between mean (geometric) counts from each method (Earle and Perez over Thick film method). (c) Comparison of Thick Film with Thin Film methods (after averaging thick Film method across all raters). X axis: Mean (geometric) averaged across both methods. Y axis: Relative difference between mean (geometric) counts from each method (Thick film over thin film method).

Most relative differences are between a 50% increase in counts and a one third reduction (which is a difference of the same size but in the opposite direction on a multiplicative scale). Points appear to be scattered relatively evenly above and below the line of no relative difference. Earle and Perez gave counts an average of 4.7% lower than the thick film method, 95%CI 13.4% lower to 4.9% higher. Since the 95%CI includes both higher and lower values, we do not have any evidence that any such bias exists (p = 0.32). Nor is there any evidence of changing level of bias with parasite count (p = 0.45 from regression on count).

### Agreement between counts from Earle and Perez and the thin film method (Figure 4b)

Here there is evidence of bias (p = 0.0002), as demonstrated by the fact that most points lie below the equal line, and therefore demonstrate that Earle and Perez tends to give lower counts than the thin film method. On average, Earle and Perez gives counts that are 31% lower than the thin film method (95%CI 18% to 42% lower). There is no evidence that the extent of this bias changes with parasite count (p = 0.10).

### Agreement between counts from the thick film and the thin film method (Figure 4c)

A similar result is found when the thick and the thin film methods are compared (Figure 4c). Again, there is evidence of a systematic bias (p = 0.003). The thick film method gives counts an average of 26% lower than the thin film method (95%CI 10% to 39%, p = 0.003). There is no evidence that the extent of this bias changes with parasite count (p = 0.10).

## Discussion

### Comparison of methods

There is little or no bias in counts between Earle and Perez and the thick film method (p = 0.32). However, both these methods give counts that are around 30% lower on average than those obtained from the thin film method. Earle and Perez gives counts an average 31% lower than the thin film method (95%CI 18% to 42% lower, p = 0.0002), while the thick film method (based on white cell count) gives counts an average of 26% lower (95%CI 10% to 39%, p = 0.003). This was as expected from previous work on estimation of malarial parasitaemia. For example, Dowling and Shute [7] compared parasite counts obtained by examination of thin and thick blood films and concluded that parasite losses of 60 to 90% occurred with thick films, whereas since thin films are fixed after drying and before staining, they assumed no significant loss of parasites during staining. Bejon *et al *[8] confirmed detachment or lysis of parasites from thick films during staining by detecting *Plasmodium falciparum *DNA in the reagents used to stain the films. Using a series of parasite dilutions, they found that thick films tended to measure parasite densities around one log lower than the number calculated to be in the dilution and this did not vary by microscopist. Thus, although thick films are more sensitive for parasite detection than thin films (due to the fact that they examine a larger volume of blood) they consistently underestimate the level of parasitaemia. Comparison of the thick film (wbc) method and the thin film (rbc) method by O'Meara *et al *[9] showed that measurements from the thin film averaged approximately 30% higher than the total mean, and parasitaemia from the thick film averaged 10% lower than the total mean (p = 0.0011). One defect in the wbc method is that it assumes that wbcs are evenly distributed in the film, but O'Meara *et al *showed that they were much less uniformly distributed than the parasites. They also confirmed previous reports that up to 60% of parasites were obscured in the thick film or lost during the process of red cell lysis and parasite staining. In this study, zero to 65% fewer parasites were counted in the thick compared to the thin film.

Interestingly, loss of white cells from thick films appears to be much lower than loss of parasites. Dowling and Shute [7]reported an average loss in routine staining of only 8%, whilst Earle and Perez [2]measured white cell counts by haemocytometer and by direct counting of cells in a given volume of blood on a thick film and found haemocytometer counts to be only 2.3% higher than the thick film counts.

The total white blood cell count for the samples examined in our study ranged from 2470 to 14,200 with a mean of 6,359/μL. If parasitaemia had been calculated with reference to a standard figure of 8,000/μL, significant under- and overestimates of parasitaemia would have been made using method 2. Greenwood and Armstrong [10] found this to be a disadvantage when comparing diagnosis by thick versus thin films. They compared parasitaemia obtained from a knowledge of the red cell count and the level of infection in red blood cells in a thin blood film with two techniques based on thick films. In the first, parasitaemia was calculated by counting the number of parasites present per white cell in a thick film and multiplying it by 8,000, regarded as an average white blood cell count per microlitre. In the second, the number of parasites per high power microscope field was counted and the parasitaemia calculated using this figure and the volume of blood assumed to be present in a high power field. They found the second method to be more accurate (compared to thin film examination) and felt that it was due to variability in the volume of blood used to prepare thick films being less than the variability in white blood cell count in the population they studied. Once the lack of an accurate white blood cell count is overcome, as in our study, the reliability of the thick blood film method improves.

### Comparison of raters

This study has shown differences between methods using the same microscopy staff, but reader technique itself clearly contributes to the accuracy of parasitaemia estimates.

The inter-rater reliability appears to be fairly similar for Earle and Perez and the thick film method, although the estimated reliability is slightly better for the thick film method. For Earle and Perez, 95% of counts from different raters will lie between 48% of the geometric mean count across all raters, for the specified sample, and 2.10 times this count. For the thick film method, 95% of counts from different raters will lie between 54% of the geometric mean count across all raters, for the specified sample, and 1.84 times this count. The intra-rater reliability appears to be much better using Earle and Perez, than using the thick film method. However, this is based on assessment of one rater alone, and may be because she used two different techniques to perform the thick film method. Another possible explanation is that the distribution of white blood cells is much less uniform than that of the parasites [9], which would affect the white cell method but not the Earle and Perez method.

Killian *et al *[11] examined inter rater variability in the results of malaria microscopy in epidemiological studies using 711 thick blood films re-read by four experienced microscopists. Parasite density was calculated by counting the number of trophozoites in 100 oil immersion fields and multiplying by 4 to give parasites per microlitre, assuming a blood volume of approximately 0.25 μL per 100 microscope fields (as previously used by Dowling and Shute [7] and by Greenwood and Armstrong [10]). Variability around the mean difference continuously decreased with increasing parasite density. When two parasite counts for the same slide were compared they found considerable variability, with one reading being 0.12 to 10 times the other. There was significantly less variability at parasite densities above 5000/μL (0.2 to 3.6 times) but it was still substantial.

O'Meara *et al *[12] examined sources of variation in parasite density measurements using the same technique as our method two. Overall, for variation between readers, they stated that discrepancies in parasite densities reported by experienced clinic microscopists decreased with increasing mean density and trends were similar for *P. falciparum *and for *Plasmodium vivax *when they were considered separately. They also commented that if each count had been multiplied by a uniform approximation of the white cell count instead of the actual figure, this relationship between density and discrepancy would have been obscured. These authors felt that when agreement between readers is required, identical technique, consistently applied, may be more important than increasing the number of microscope fields read. They cited other causes of errors in density measurements as slide quality and the random distribution of parasites and white cells within the blood film.

O'Meara *et al *[9] sent slides from 35 blood samples to 27 reference readers from 13 countries and asked them to record the density of asexual and sexual parasites of each species seen on the slide. They found a significant inverse correlation between discrepancy among microscopists and mean parasite density. Furthermore, they suggested that random chance in the selection of fields to examine may play a large part in reader discrepancy, especially with low parasitaemia. In their study, for the thick film (wbc) method the parasite counts were all derived using the standard approximation of 8,000 wbcs per μL and the group mean for a sample was scaled by the group mean, then plotted against the mean density for the sample. For all samples the deviation from the mean decreased as the number of reference wbcs increased. There was a significant negative correlation between the magnitude of the residuals and the number of wbcs counted for each measurement. In order to improve agreement among readers O'Meara *et al *suggested that a uniform counting protocol should be used and the number of white cells or red cells counted should be increased [9]. Both precision and accuracy could be improved if the number of parasites per given volume could be counted, eg with the aid of a grid.

The laboratory personnel in this study were all experienced malaria microscopists and the results may be different with non-specialists. For example, in UK practice, diagnostic laboratory staff are less familiar with thick blood films than with thin films for malaria diagnosis (UK NEQAS, personal communication, 2008). However, the preparation of the standard panel is being undertaken by staff experienced in blood film examination, so the conclusions of this study remain applicable in that context. The thin film method, whilst closer to the true parasite count, is not feasible at parasite counts below 500 parasites per microlitre, so the thick film method (method two) has been selected as both reproducible and practical for the project because it gave slightly better inter-rater agreements, and was easier to perform, and is generally better known by malaria microscopists. In the long term, recombinant HRP2 could be used as a reproducible, stable and standardized substitute for *P. falciparum *infected blood in some parts of an RDT product assessment programme and for lot testing of purchased RDT devices, though it cannot completely supplant the use of infected blood. Until rapid, reproducible, quantitative PCR for malaria is widely available, microscopy will remain the method of choice for the determination of malarial parasitaemia, but it must be applied by skilled operators using standardized techniques, such as those assessed in this study.

## Conclusion

The thin film method gave results closer to the true parasite count but is not feasible at a parasitaemia below 500 parasites per microlitre.

The thick film method (method two) counting parasites against 500 white blood cells and adjusting for the actual white cell count in a given patient, gave slightly better inter-rater agreements than the Earle and Perez method, was easier to perform and thus was selected for use in the RDT project. Until rapid, reproducible, quantitative PCR for malaria is widely available, microscopy will remain the method of choice for the determination of malarial parasitaemia, but it must be applied by skilled operators using standardized techniques, such as those assessed in this study.

## Competing interests

The authors declare that they have no competing interests.

## Authors' contributions

KB was the reference microscopist, participated in drafting the manuscript and supervised the collection of samples and pre-training of the microscopists in Cambodia and the Philippines, DB conceived the study and participated in its design and coordination, PC supervised all aspects of the study carried out in the United Kingdom and the drafting of the manuscript, JB supervised the microscopists in the United States and returned the results to the reference microscopist for analysis, SI organized the collection of samples in Cambodia and was the overall supervisor at this site, SY oversaw the microscopists and the correlation of the results in Cambodia, JL supervised the microscopists in the Philippines and correlated the data for the reference microscopist, HW performed all the statistical analysis for the study. All authors read and approved the final manuscript.
